# Single-operator cholangioscopy and electrohydraulic lithotripsy for the treatment of Mirizzi syndrome

**DOI:** 10.1016/j.amsu.2021.01.031

**Published:** 2021-01-23

**Authors:** Gustavo Salgado-Garza, Pamela Hernandez-Arriaga, Mauricio Gonzalez-Urquijo, José Antonio Díaz-Elizondo, Eduardo Flores-Villalba, Javier Rojas-Méndez, Mario Rodarte-Shade

**Affiliations:** a Tecnologico de Monterrey, Escuela de Medicina y Ciencias de la Salud, Dr. Ignacio Morones Prieto, O 3000, 64710, Monterrey, Nuevo Leon, Mexico; b Tecnologico de Monterrey, Escuela Nacional de Ingeniería, Departamento de Ciencias Clinicas, Hospital Zambrano Hellion, Batallon de San Patricio, 112. Col. Real de San Agustin, Monterrey, 66278, Mexico

**Keywords:** Mirizzi syndrome, Peroral cholangioscopy, Lithotripsy, Endoscopic retrograde cholangiopancreatography, Cholelithiasis

## Abstract

**Introduction:**

Mirizzi syndrome is an infrequent complication of long-standing cholelithiasis. Extrinsic compression of the common hepatic duct is usually caused by an impacted stone in Hartmann's pouch or cystic duct resulting in the development of cholecystobiliary fistula. This syndrome is classified based on the presence and severity of cholecystobiliary fistula. Mirizzi syndrome is challenging to diagnose preoperatively and may require complex biliary surgical procedures for resolution.

**Results:**

We present three patients with Mirizzi syndrome with different clinical presentations. All were successfully treated by cholangioscopy with electrohydraulic lithotripsy. Endoscopic treatment is a safe alternative with a high success rate. Single-operator cholangioscopy combined with lithotripsy has been shown to have a 90–100% success rate in the treatment of biliary stones.

**Conclusion:**

Herein, we present our experience treating Mirizzi syndrome with single-operator cholangioscopy guided electrohydraulic lithotripsy. Difficult management of Mirizzi syndrome has led to research of new treatment options to minimize the risk of high-rate complications. Single-operator cholangioscopy in combination with laser lithotripsy is an adequate and safe alternative for the treatment of this condition.

## Highlights

• Mirizzi syndrome has no current gold-standard for treatment. Avoiding damage to the biliary tract has been a challenge.
• Adequate management is achieved with direct visualization aided of cholangioscopy and lithotripsy.
• Mirizzi syndrome can have different clinical presentations.

## 1 Introduction

Both symptomatic cholelithiasis and acute cholecystitis encompass some of the most common pathologies treated by surgeons. Mirizzi syndrome is a rare complication of gallstone disease, which is found only in 1.3% of all patients undergoing cholecystectomy regardless of the cause [1]. Therefore, Mirizzi syndrome is the rarest among uncommon complications such as gallstone ileus and cholecystoenteric fistulas.

Mirizzi syndrome is characterized by compression of the common hepatic duct (CHD) by a gallstone in either the Hartmann's pouch or cystic duct resulting in the development of cholecystobiliary fistula. The clinical manifestations are of obstructive jaundice, fever, and right upper quadrant pain. Due to the atypical nature of the disease, Mirizzi syndrome is seldomly diagnosed preoperatively [2].

We present our experience in successfully treating Mirizzi syndrome through an endoscopic approach with single-operator cholangioscopy guided electrohydraulic lithotripsy, in an academic medical center. A description of Mirizzi syndrome and its treatment options are also discussed.

The work has been reported in line with the PROCESS guidelines [3]. The work is registered in ClinicalTrials under ID number NCT04672902.

## 2 Case presentations

### 2.1 Case 1

A 34-year-old female with no prior medical history arrived at the emergency department with a 2-month history of intermittent right upper quadrant pain. Physical examination was unremarkable except for localized abdominal tenderness at the right upper quadrant and generalized jaundice. Laboratory workup upon admission revealed total bilirubin 2.93 mg/dL, conjugated bilirubin 1.69 mg/dL, and unconjugated 1.24 mg/dL, aspartate aminotransferase (AST) 279 U/L, alanine aminotransferase (ALT) 990 U/L and alkaline phosphatase (AP) of 291 U/L, GGT of 338 U/L with all other parameters within normal ranges.

**Fig. 1:**
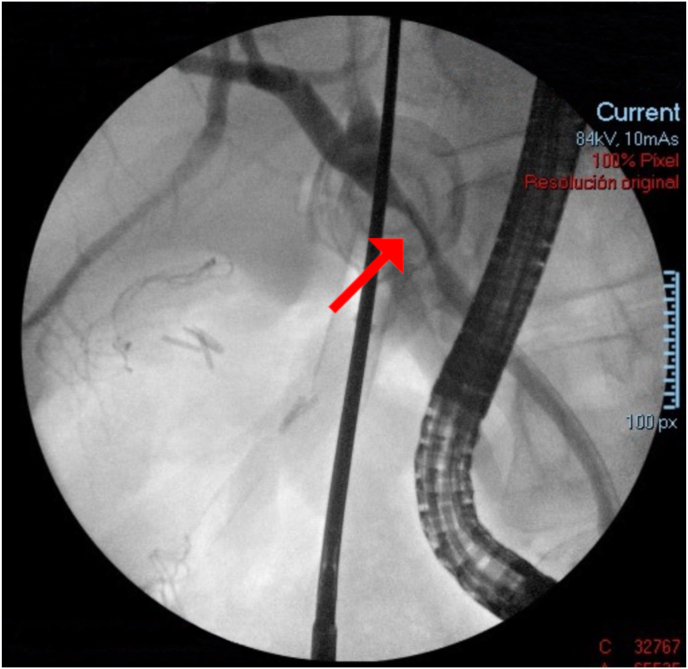
Intraoperative ERCP showing stenosis of CBD. Red arrow showing stenosis of CBD. ERCP = endoscopic retrograde cholangiopancreatography, CBD = common bile duct. (For interpretation of the references to colour in this figure legend, the reader is referred to the Web version of this article.)

The patient was transferred to the operating room to perform a laparoscopic cholecystectomy. Intraoperatively, dense adhesions, extensive acute, chronic inflammation, and an impacted stone were found in the Hartmann pouch, causing extrinsic compression of the common bile duct (CBD). Adequate dissection of the cystic duct was impossible to achieve, so a fenestrated partial cholecystectomy was completed. An intraoperative ERCP was performed to visualize the previously seen stenosis of the CBD (Fig. 1). Bipolar electrohydraulic lithotripsy with a 1.9 Fr Autolith™ (Boston Scientific; Massachusetts, USA) was performed by an attending surgeon with advanced endoscopic training, with energy levels of 12 W and a guidewire, clearing the CBD. Finally, a plastic biliary endoprosthesis was placed. The patient was discharged on postoperative day (POD) 2 with a final diagnosis of Mirizzi Syndrome Type I.

Two months after the first admission, the patient arrived at the emergency department once more with an identical clinical picture than the first episode. A computerized tomography (CT) scan connoted the migration of the biliary endoprosthesis. An ERCP was performed using SpyGlass® with Autholith™ (Boston Scientific; Massachusetts, USA) and the prosthesis was indeed found in the second part of the duodenum and the stone was now located at the distal bile duct. Electrohydraulic lithotripsy using a Holmium laser at 12 W was performed and 70% of stone fragments were removed. A plastic biliary endoprosthesis Tannenbaum® by Cook Medical (Bloomington, Indiana, USA) was placed.

**Fig. 2:**
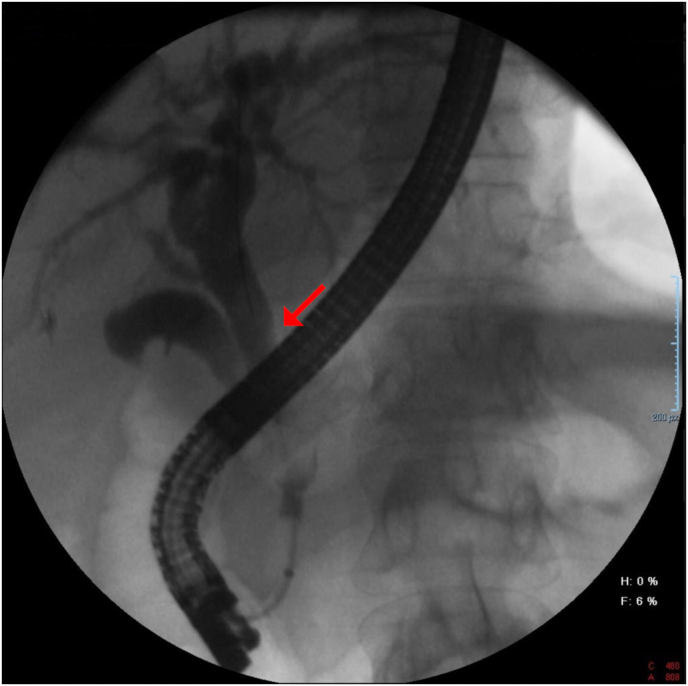
Final ERCP with normal passage of contrast material, without filling defects. Adequate passage of contrast as depicted with a red arrow. ERCP = endoscopic retrograde cholangiopancreatography. (For interpretation of the references to colour in this figure legend, the reader is referred to the Web version of this article.)

Three months after the initial episode, another peroral cholangioscopy using SpyGlass® (Boston Scientific; Massachusetts, USA) was performed. A satisfactory fragmentation of remaining gallstones by lithotripsy was achieved. A control ERCP was taken with standard passage of contrast material to the duodenum. (Fig. 2). The plastic biliary endoprosthesis was removed eight weeks after her last ERCP. The patient is currently asymptomatic a year after her initial procedure, with normal liver function tests.

### 2.2 Case 2

A 74-year-old male with a past medical history of hypertension, arrived at the emergency department, referring to persistent fever and lower limb weakness for the past two weeks. Physical examination was otherwise unremarkable, with no abdominal tenderness nor jaundice. The patient was admitted for the workup of fever of unknown origin. Laboratory studies included complete blood count, liver function tests among others. Relevant laboratory results are as follows; total bilirubin 1.30 mg/dL, conjugated bilirubin 0.71 mg/dL, unconjugated 0.59 mg/dL, AST 121 U/L, ALT 250 U/L, alkaline phosphatase 747 U/L, GGT 740 U/L with other accompanying values were found within normal ranges for the patient.

**Fig. 3:**
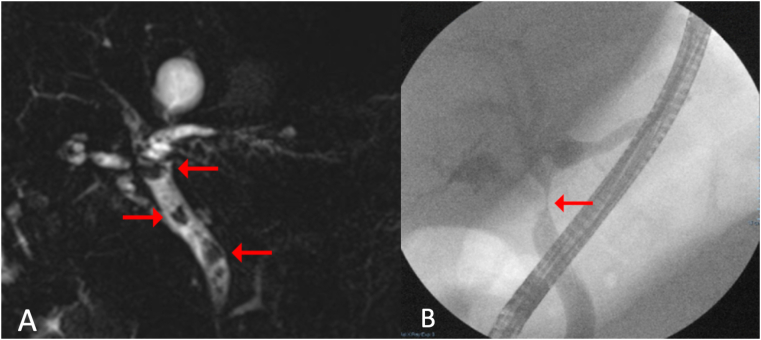
A. Magnetic resonance cholangiopancreatography with intrahepatic biliary duct dilation of 6.7 mm and CBD dilation of 8.4 mm, showing multiple stones. Arrows in the image point towards biliary stones. **B.** ERCP showing intrahepatic and extrahepatic dilation, CBD with a diameter of up to 12 mm, and stenosis suggesting extrinsic compression. Red arrow points towards the site of stenosis. ERCP = endoscopic retrograde cholangiopancreatography, CBD = common bile duct. (For interpretation of the references to colour in this figure legend, the reader is referred to the Web version of this article.)

An abdominal CT was performed showing acute calculous cholecystitis complicated with a dilated CBD. A complementary MRCP unveiled a CHD of 6.7 mm and CBD dilation of 10 mm, with stenosis at the CHD. Furthermore, abrupt stenosis of the ampulla was reported. An ERCP was performed, and a wide sphincterotomy was accomplished (Fig. 3). A solitary stone was found eroding the bifurcation of the left and right hepatic duct and the beginning of the CHD. Subsequently, using single-operator cholangioscopy technology, SpyGlass®, the already seen gallbladder stone, was found eroding the CHD wall. Single operator cholangioscopy aided with bipolar electrohydraulic lithotripsy using a 1.9Fr Autolith™ (Boston Scientific; Massachusetts, USA) device was performed by an attending surgeon with advanced endoscopic training, with energy levels at 15 W. Approximately 30% of the stone eroding the CHD was able to be fragmented. Nevertheless, the remainder of the gallstone remained inside the fistulized gallbladder. Finally, a 10 cm x 10 Fr plastic biliary stent was left behind, guided towards the left hepatic duct. The patient was discharged asymptomatic with normal liver function tests on POD 14 and with a final diagnosis of Mirizzi syndrome type III.

Due to unsuccessful complete extraction of the stone, surgical intervention was proposed; however, the patient was ruled unfit for major surgery. Six weeks after the initial admission, another ERCP was performed. Several gallstones were still visualized in the CBD. The stone left behind at the gallbladder was migrated entirely to the CHD. Using SpyGlass® and lithotripsy using a 1.9Fr Autolith™ (Boston Scientific; Massachusetts, USA), electrohydraulic lithotripsy was completed, and both hepatic ducts were cleared of stone debris. A 10-Fr., 10 cm plastic biliary prosthesis was placed due to eroded mucosa seen at the site of prior cholecystohepatic duct fistula. Biopsies were taken to exclude malignancy.

Twelve weeks after the initial ERCP, another cholangioscopy was performed to verify ductal clearance. No fistulous tracts or residual stones were seen, and the biliary stent was removed. At a one-year follow-up, the patient has remained asymptomatic.

### 2.3 Case 3

A 79-year-old female admitted to the hospital for community-acquired pneumonia presented with fever and right upper quadrant abdominal pain. Laboratory workup revealed leukocytosis of 17.6 ( × 10^9^)/L and within-range liver function test results. An abdominal CT found pneumobilia with a 3 cm gallstone inside the gallbladder. Next, an ERCP was done, and a biliary stone was found lodged in the infundibulum of the gallbladder, accompanying a cholecystoduodenal fistula. Single-operator cholangioscopy with laser lithotripsy was performed by an attending surgeon with advanced endoscopic training, for the management of this condition instead of a more invasive therapeutic approach. Laser fragmentation of the gallstone was successfully achieved, and basket retrieval of the fragments was performed. Finally, a pigtail catheter was set in the fistula tract. The patient was discharged on POD 3 with a diagnosis of Mirizzi syndrome type IIIa. Twenty weeks after the first procedure, a second ERCP was performed with findings of the cholecystoduodenal fistula's spontaneously resolution and normal biliary tracts. At one year follow up, the patient remains asymptomatic.

## 3 Discussion

Mirizzi syndrome is an uncommon complication of prolonged cholelithiasis. It occurs in 0.05–2.7% of all patients with cholelithiasis, and it is caused by extrinsic compression of the CHD, usually from a stone impacted in Hartmann's pouch of the cystic duct, which leads to the development of cholecystobiliary fistula [[4], [5], [6]].

This syndrome can be classified into five categories depending on the presence and severity of cholecystobiliary fistulization [2,7,8]. Type I lesions include those with external compression of the CBD. Type II presents a cholecystobiliary fistula with an erosion of less than one-third of the bile duct. Type III is when the fistula involves two-thirds of the duct circumference, and in type IV, there is a complete destruction of the bile duct [4,9]. Type V lesions are composed of those with a cholecystoenteric fistula and can be subcategorized in Va, without gallstone ileus, and Vb, with gallstone ileus [10]. Our patients had type I, III and III Mirizzi syndrome, respectively.

Ultrasound is usually the first study to be made in a routine investigation of biliary disease, but sensitivity for Mirizzi syndrome is very low [11,12]. The imaging modality of choice is an MRCP; this noninvasive imaging technique has a 50% diagnostic accuracy rate [11]. On the other hand, ERCP is considered the gold standard for diagnosing Mirizzi syndrome with a diagnostic accuracy of 55%–90%, with the advantage of also providing therapeutic functions such as stone extraction [4,5,8].

The inflammatory response associated with the cystic duct stones found in this syndrome poses a surgical challenge when dissecting the hepatocystic triangle [4,8]. Consensus over the optimal therapeutic approach is yet to be defined, with some authors suggesting conventional or laparoscopic cholecystectomy [10,13]. However, the latter carries a conversion rate of 41% with a rate of complications of up to 20%. Treatment modalities also depend on the severity of the syndrome. Type I is usually managed with a total or subtotal cholecystectomy. Type II requires a subtotal cholecystectomy with bile duct reconstruction. Type III and IV require a bilio-enteric anastomosis [8]. Therapeutic approach should be tailored on a case-to-case basis. In our first case, we achieved a partial cholecystectomy, recurring to a complementary endoscopic treatment to eliminate the impacted stones on the biliary tree.

Difficulties in the management of Mirizzi syndrome has led to the search for new treatment options to minimize the risk of complications. Endoscopic treatment is a safe alternative with a high success rate [8]. For larger stones (>2 cm), the suggested treatment is mechanical, electrohydraulic or laser lithotripsy, along with sphincterotomy and balloon dilation [6,8,14,15]. Mechanical lithotripsy is usually effective in 80–90% of the cases, however in our cases, a stone in the cystic duct made this method unachievable, so we used electrohydraulic and laser lithotripsy with direct visual control. In our patients, this was achieved by using SpyGlass® cholangiopancreatoscope and AutoLith laser lithotripter. These techniques have the advantage of providing direct visualization of the bile ducts, enabling a single physician to diagnose and perform the therapeutic intervention in a single procedure [13,16]. Single operator cholangioscopy, combined with lithotripsy, has been shown to have a success rate of 90–100% in the treatment of biliary stones [5,8,17]. Despite the high efficacy of this technique, around 10–15% of patients will require multiple sessions [17,18]. In our first two cases, numerous laser lithotripsy sessions were necessary for adequate fragmentation of the stones.

## 4 Conclusion

The importance of preoperative diagnosis of Mirizzi syndrome to avoid bile duct injuries or lesions to adjacent organs should be exalted. Difficult management of Mirizzi syndrome has led to research of new treatment options to minimize the risk of the aforementioned complications. Single-operator cholangioscopy, combined with laser lithotripsy, is an adequate and safe alternative for the treatment of this condition with a success rate of 90–100% and lower complication rates. Further research in larger cohorts comparing surgical techniques for management of Mirizzi Syndromes is needed before consensus can be achieved.

## Consent

Written informed consent was obtained from the patients for publication of this case series and accompanying images. A copy of the written consent is available for review by the Editor-in-Chief of this journal on request.

## Declaration of competing interest

None.

## Registration of Research Studies

1. Name of the registry:

Treatment of Mirizzi Syndrome.

2. Unique Identifying number or registration ID:

NCT04672902.

3. Hyperlink to your specific registration (must be publicly accessible and will be checked):

https://clinicaltrials.gov/show/NCT04672902.
